# Bisphosphonates as a therapeutic choice for multifocal epithelioid hemangioma of bone: A case report

**DOI:** 10.1097/MD.0000000000018161

**Published:** 2019-11-27

**Authors:** Lizhi Tang, Guangwen Chen, Qin Wang, Jobin John, Chunyan Lu

**Affiliations:** a Department of Endocrinology and Metabolism, West China Hospital of Sichuan University; b Department of Radiology, Sichuan Provincial People's Hospital, Chengdu, China.

**Keywords:** bisphosphonates, epithelioid hemangioma, metabolic bone markers, zometa

## Abstract

**Rationale:**

Epithelioid hemangioma (EH) of bone is an intermediate vascular tumor that can be locally aggressive. The optimum management of multifocal EH of bone is not well delineated. We described our experience treating one patient with multifocal EH of bone in an effort to document the effect of bisphosphonates in bone EH.

**Patient concerns:**

In this report, a 53-year old male patient presented with back pain which was initially been diagnosed of multiple bone metastatic carcinoma by 18F-FDG PET/CT scan and bone scintigraphy.

**Diagnosis:**

CT-guided bone biopsy of ilium indicated that puncture tissue had irregular hyperplasia of thick and thin-walled blood vessels, immunohistochemistry revealed positive staining for CD31 and CD34, negative for CAMTA-1, PCK and EMA, which confirmed the diagnosis of multiple EH.

**Interventions:**

The patient was treated with 4 times of intravenous Zometa (zoledronate, 4 mg each time) with average three-month interval. Bone metabolic markers including serum bone specific alkaline phosphatase (BALP) and type I collagen cross-linked C-terminal telopeptide (CTX) levels were closely monitored before and after use of bisphosphonates each time.

**Outcome:**

BALP and CTX were significantly lowered following intravenous Zometa and the back pain improved with integrated therapy including bone graft fusion internal fixation surgery and vertebroplasty.

**Conclusions:**

EH of multiple bones responded favorably to intravenous Zometa with improvement of bone metabolic markers. After 1 year on follow-up, the patient was doing well with no significant pain. We suggest that bisphosphonates should be considered in the treatment of multifocal osteolytic EH of bone.

## 1 Introduction

Epithelioid hemangiomas (EH) are rare, locally aggressive neoplasms comprising of cells with endothelial phenotype and epithelioid morphology.^[1]^ Most common location of EH is soft tissues, followed by bone as the second most common location.^[2]^ According to World Health Organization classification of bone tumors (4th edition), EH of bone is classified as intermediate neoplasms.^[1]^ In EH of bone, long tubular bones are the most common locations, followed by the short tubular bones of the distal lower extremity, the flat bones, vertebrae and the small bones of the hands.^[3,4]^ Solitary lesions are the most common. However, multifocal lesion has been reported in 18% to 25% of cases, most commonly involving the same bone or same extremity.^[1,4,5]^ Most cases occur between 20 to 60 years of age.^[4]^

Bisphosphonates has been reported in treating hemangioma and epithelioid hemangioendothelioma (EHE).^[6–8]^ However, little is known about the use of bisphosphonates in bone EH. We herein report our experience with a patient suffering from multifocal EH of the bone which responded favorably to intravenous Zometa showing improvement of pain and bone metabolic markers. After 1 year on follow-up, the patient was doing well with no significant pain or swelling. The clinical, radiographic and pathologic aspects are also described.

## 2 Case report

This 53-year-old retired man presented with bilateral thigh, buttocks and back pain for 2 months prior to admission. The pain was intermittent, which became worse at night seriously affecting his sleep. He also felt back weakness, could not keep his back upright while walking. He had a history of splenectomy 11 years ago, due to moderate anemia caused by an unexplained splenomegaly. He had no bone pain or neurological damage at that time. Postoperative pathological results showed only splenic hemolytic enlargement accompanied by iron-containing nodule formation and focal infarction, with no lymphoma or other tumor-like changes. We reviewed the pathological results of spleen and no epithelioid hemangioma like changes was found.

**Figure 1:**
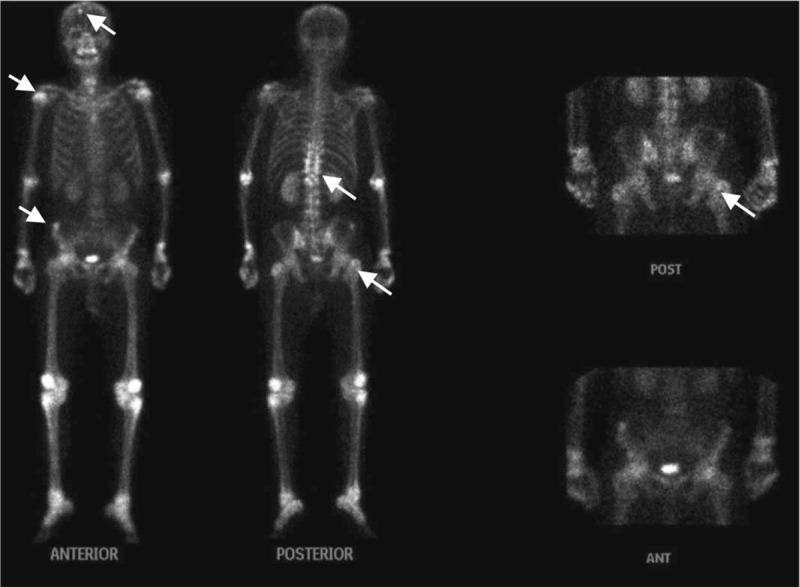
^99m^Tc-MDP bone scintigraphy showed multiple lesions of increased activity in spine, pelvis, skull, scapula and humerus.

**Figure 2:**
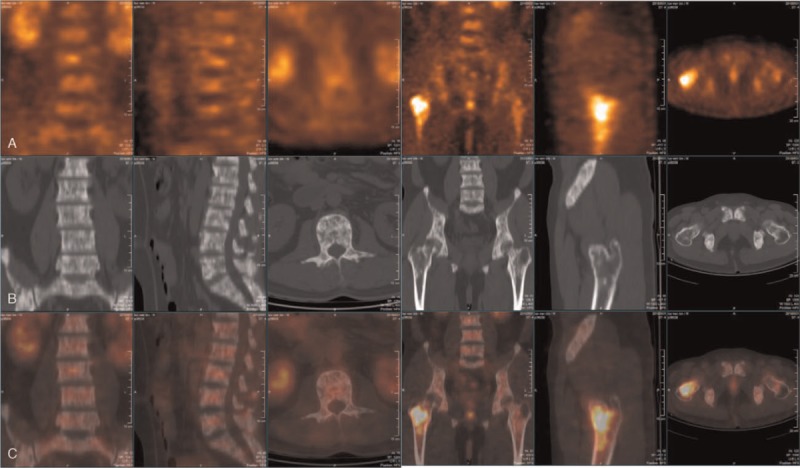
Multiple hypermetabolic skeletal lesions on ^18^F-FDG-PET/CT. Bone lesions involving the spine, pelvic bones, both humeri and both femori without any other extraosseous suspicious activity. The CT images showed diffuse, patchy osteoblastic lesions were scattered throughout the skeleton with increased uptake of ^18^F-FDG. Mixed blastic and lytic lesions with intense ^18^F-FDG uptake (row A: PET; row B: CT; row C: fusion image) were found with a maximum standard uptake.

**Figure 3:**
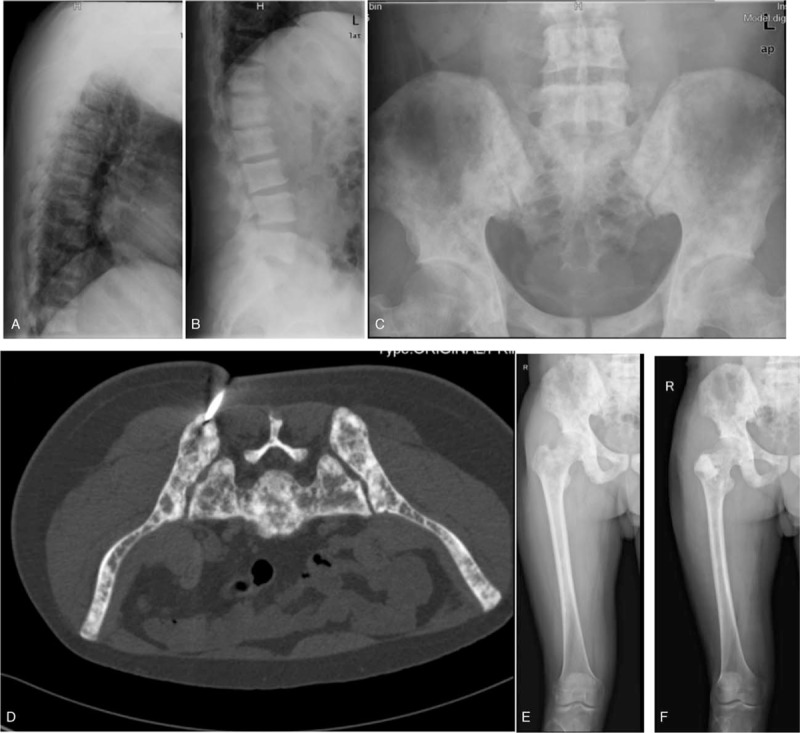
Bone imaging findings before Zometa therapy. (A and B) Lateral radiographs of the thoracic (A) and lumbar (B) vertebra showed flattening of the vertebral body, increase of bone density and widening of intervertebral space. (C) Pelvic plain radiographs showed multiple lesions with increased and decreased bone density. (D) CT-guided iliac bone biopsy, diffuse high and low mixed density lesions of the ilium and sacrum can be seen, with multiple bone destruction. (E) Prior to biopsy of the upper right femur, a general increase of bone density from the pelvis to the upper femur, with mixed high and low density. (F) After bone biopsy of the upper segment of the right femur, a cement filling shadow at the biopsy can be seen.

**Figure 4:**
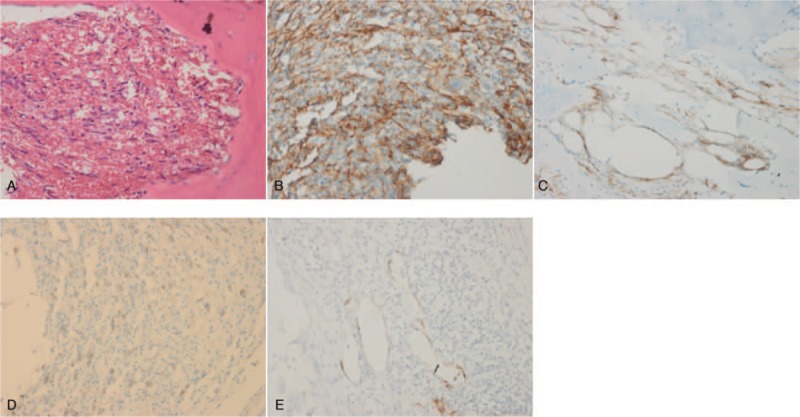
Pathological and immunohistochemical findings of femur biopsy. (A) Hematoxylin-eosin stain showed irregular hyperplasia of thick and thin walled blood vessels. Immunohistochemistry revealed positive staining for CD31 (B), CD34 (C), ERG (D), and factor VIII (E). All presented as original magnification ×400.

**Figure 5:**
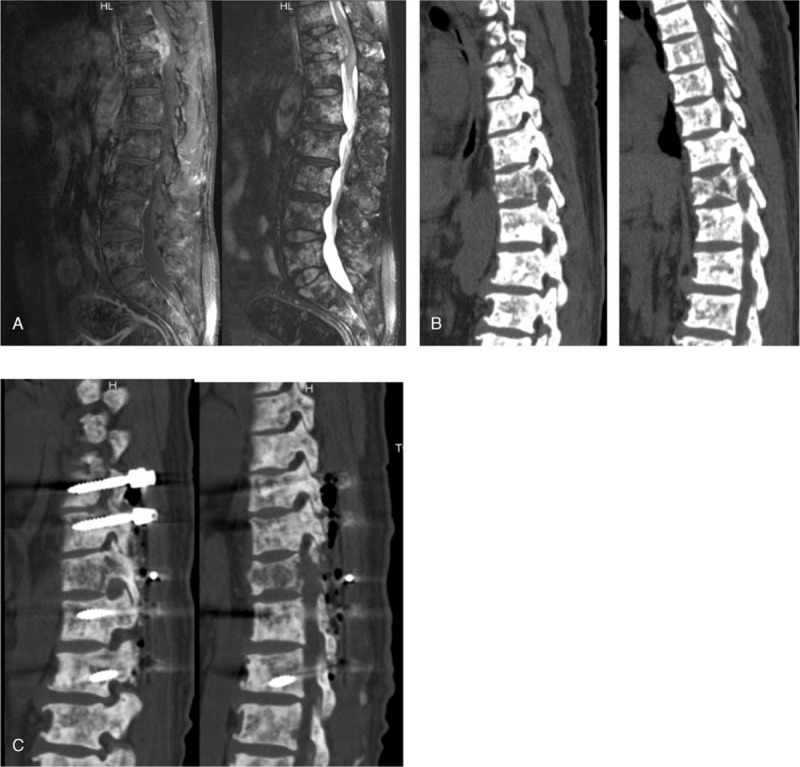
Imaging changes before and after T11 vertebral fracture leading to spinal cord compression and incomplete paralysis. (A) MRI findings showed that the lumbar vertebra was flattened and wedged to different degrees, the T2WI signals of the vertebra and the attachment were uneven and presented diffuse mixed signals of high and low levels, and the fatty inhibition T1WI enhanced scan sequence showed obvious uneven enhancement of the thoracic vertebra 11 and 12. Three-dimensional CT reconstruction, before surgery (B), 10 days after surgery (C).

After relevant examination, ^99^mTc-MDP bone scintigraphy showed multiple metabolism enhancements of the multiple bones; multiple bone metastases were suspected (Fig. 1). ^18^F-FDG-PET/CT showed multiple hypermetabolic skeletal lesions, bone metastasis or metabolic bone disease was suspected (Fig. 2). X-ray showed that bone density of cervical vertebrae, thoracic vertebrae, lumbar vertebrae, skull, pelvis, bilateral scapula, bilateral clavicle, bilateral ribs, bilateral humerus and upper femur were generally increased, with multiple high-density and low-density nodules; metabolic bone disease was suspected (Fig. 3). Thereafter we performed CT-guided biopsy of the left ilium and right femoral trochanter area, pathological findings indicated that puncture tissue had irregular hyperplasia of thick and thin walled blood vessels; multiple epithelioid hemangioma was considered (Fig. 4). The patient had elevated serum bone specific alkaline phosphatase (BALP) and type I collagen cross-linked C-terminal telopeptide (CTX) levels. He was treated with 2 times of intravenous Zometa (zoledronate; Novartis Pharmaceuticals, Basel, Switzerland, 4 mg) with three-month interval, 4 mg each time, which lowered BALP and CTX levels significantly and provided control of the patient's bone pain (Table 1). However, the effects were short lived with the pain returning 1 month after the second course of intravenous Zometa; the patient suffered paraplegia and was admitted under the department of orthopedics. Three-dimensional CT suggest multiple bone destruction of the spine and its attachments, T11 compression fracture, with diagnosis of thoracic 11–12 tumor with paraplegia (Frankel A). Post-lumbar thoracic 11–12 tumor resection, spinal canal decompression, bilateral spinal nerve release, bone graft fusion internal fixation was performed (Fig. 5). The postoperative pathological results still suggested the diagnosis of epithelioid hemangioma of bone. His pain was controlled for several months. Unfortunately, three months after the surgery, the bone pain aggravated again, which was more obvious at night, with difficult of walking upright, he can only stand with the support of his hands could not stand upright and his activity time was shortened. The patient was given another 2 times of Zometa with three-month interval, with significant improvement of bone metabolic markers (Table 1). He also underwent T11 vertebroplasty and the bone pain was significantly relieved. The patient was followed up every 2–3 months to check for any relapse, he was doing well with no significant pain after one year follow up.

**Table 1:**
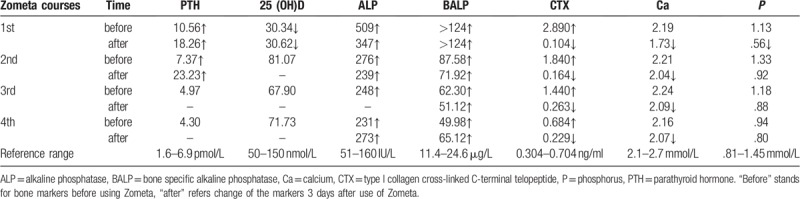
The change of metabolic bone markers responded to Zometa.

This case report was approved by the Ethics Committee of the West China Hospital of Sichuan University, Chengdu, China, and the patient has provided informed consent for publication of the case.

## 3 Discussion

Epithelioid hemangioma of bone is now classified as intermediate bone tumor according to 2013 World Health Organization classification and it can cause lytic changes within the bone leading to fractures and instability.^[3,9]^ Additionally, the tumor can extend into adjacent soft tissue and cause spinal cord compression. When instability and/or cord compression are present, appropriate surgical treatment should be instituted. However, because EH can behave in an indolent fashion, patients with non-aggressive tumors should be followed clinically and radiographically for signs and symptoms of instability and/orspinal cord compression.

Tumors arising from the vascular elements of bone has been divided into three classification, benign (hemangioma), intermediate (epitheliod hemangioma), or malignant (epitheloid hemangioendothelioma and angiosarcoma).^[9]^ Hemangioma of bone is a benign tumor composed of capillary-like blood vessels of small or large caliber. Hemangioma affects the vertebral body and shows a coarsened vertical trabecular/corduroy pattern, giving a “polka-dot” appearance on cross-sectional imaging and due to the prominent fatty stroma it shows relatively hyperintense signal on both T1- and T2-weighted images.^[3]^ Radiographs of EH demonstrate a well-defined occasionally expansile radiolucent lesion. On MRI, they are heterogeneous but predominately T2-weighted hyperintense, similar to other vascular tumors.^[1]^ In our case they are isointense or slightly hyperintense to skeletal muscle on T1-weighted sequences. Although there are some differences regarding the radiograph manifestation, the confirmation diagnosis between EH and hemangioma rely on pathology findings.

Epithelioid hemangioma cannot be distinguished from epithelioid hemangioendothelioma (EHE) based solely on imaging, even histological differentiation can be challenging. EHE is a low- to intermediate-grade malignant neoplasm of endothelial differentiation.^[5,9]^ Radiologically, EHE produces geographic bone destruction, which in general, tends to grow in a more infiltrative pattern. While histological evaluation of EH will show more mature vessel formation and more lobulated growth pattern than EHE.^[3]^ It is important to distinguish EH from EHE because more aggressive treatment is required for EHE as it has a higher propensity for multimodality and distant spread. Multiple bones were affected in our case, EHE was initially suspected, pathological findings showed irregular hyperplasia of thick and thin walled blood vessels, more mature vessel formation confirmed the diagnosis of EH. Recently, the identification of a WWTR1-CAMTA1 gene fusion that is present in EHE, but not in EH, has allowed definitive distinction between these overlapping entities.^[5]^

Previous studies have reported that EH can successfully be treated with curettage or marginal en bloc excision. The prognosis is excellent, although the recurrence rate has been quoted to be 9%.^[9]^ Neilsen reported their experience treating 50 cases with EH, most of their patients were treated with curettage.^[4]^ Three of 50 received radiation treatment; radiation was given to help prevent another recurrence.^[4]^ They suggest that EHE should be widely excised and although the prognosis is good, a significant number of cases metastasize and prove to be fatal. These differences in treatment and prognosis emphasize the importance of accurately distinguishing between these benign and malignant neoplasms.

Bisphosphonates are potent in inhibiting osteoclast activity and promoting apoptosis, which are widely used for the treatment of osteoporosis and osteolysis diseases with minor adverse side effects. However, there was little experience about treatment for multifocal lesions with multiple bones involved. Bisphosphonates has been reported in treating hemangioma and EHE of bone.^[6–8]^ One report described an old man with unicentric grade 1 EHE of the bone that favourably responded to intravenous pamidronate as a single agent, the patient was in complete remission after 6 years of follow-up.^[6]^ Another case reported a healthy young man presented with a painful osteolytic lesion at the L2 vertebrae, imaging revealed multifocal spinal lesions, core needle biopsy confirmed the diagnosis of EHE, he underwent a partial L2 corpectomy, tumor resection, bone grafting, and vertebral reconstruction using a minimally invasive technique and followed by prolonged therapy with interferon and Zometa with good outcomes at 3.5 years follow up.^[7]^ In addition to its anti-osteolytic effect, bisphosphonates are currently shown to be capable of anti-angiogenesis and induction of apoptosis in tumor cells.^[10]^ Experimental evidences demonstrated that bisphosphonates can inhibit capillary tube formation and vessel sprouting by impairing endothelial proliferation and migration, as well as reducing the serum fibroblast growth factor-2 and vascular endothelial growth factor.^[11]^ However, little is known about the use of bisphosphonates in EH. We systematically analyzed the effect of bisphosphonates on the change of bone metabolic markers. Our result indicated that bisphosphonates can significantly lower the bone turnover markers and improve prognosis of the patients.

PET/CT findings of the EH are rarely reported before.^[12,13]^ Bone metastasis had been suspected by both ^99m^Tc-MDP and ^18^F-FDG PET/CT at the very beginning, suggest that the difficulty in differential diagnosis regarding multiple lesion of EH and metastatic carcinoma. ^99m^Tc-MDP activity are less extensive compared with ^18^F-FDG uptake, indicating mild osteoblastic activity of the lesions. We presented and compared the imaging manifestation of EH on the plain radiograph, ^99m^Tc-MDP bone scan and ^18^F-FDG PET/CT, which might help the differential diagnosis of multiple hypermetabolic bone lesions from imaging aspects in the future.

The patient was followed up every 2 to 3 months to check for any recurrence, he was doing well with no significant pain after one year follow up. More researches are needed to investigate the effect of bisphosphonates in the treatment of osteolytic epithelioid hemangioma of bone, especially with multifocal lesions.

## Author contributions

**Conceptualization:** Lizhi Tang, Chunyan Lu.

**Data curation:** Lizhi Tang, Guangwen Chen, Qin Wang, Jobin John.

**Investigation:** Qin Wang, Jobin John.

**Resources:** Guangwen Chen.

**Writing – original draft:** Lizhi Tang.

**Writing – review & editing:** Chunyan Lu.

## References

[1] Errani C Zhang L Panicek DM Epithelioid hemangioma of bone and soft tissue: a reappraisal of a controversial entity. Clin Orthop Relat Res 2012;470:1498–506. 21948309 10.1007/s11999-011-2070-0 PMC3314752

[2] Zhou Q Lu L Fu Y Epithelioid hemangioma of bone: a report of two special cases and a literature review. Skeletal Radiol 2016;45:1723–7. 27660230 10.1007/s00256-016-2482-8

[3] A. Mark Davies HD, and Steven L.J. James. Musculoskeletal Imaging. 2015.

[4] Nielsen GP Srivastava A Kattapuram S Epithelioid hemangioma of bone revisited: a study of 50 cases. Am J Surg Pathol 2009;33:270–7. 18852673 10.1097/PAS.0b013e31817f6d51

[5] Schenker K Blumer S Jaramillo D Epithelioid hemangioma of bone: radiologic and magnetic resonance imaging characteristics with histopathological correlation. Pediatr Radiol 2017;47:1631–7. 28721475 10.1007/s00247-017-3922-x

[6] Coppo P Lassoued S Billey T Successful treatment of osteolytic epithelioid hemangioendothelioma with pamidronate. Clin Exp Rheumatol 2005;23:400–1. 15971432

[7] Sebastian AS Adair MJ Morris JM Minimally invasive treatment of a painful osteolytic lumbar lesion secondary to epithelioid hemangioendothelioma. Glob Spine J 2015;5:135–9. 10.1055/s-0034-1387198 PMC4369204 25844287

[8] Yu H Qin A Could local delivery of bisphosphonates be a new therapeutic choice for hemangiomas? Med Hypotheses 2009;73:495–7. 19625131 10.1016/j.mehy.2009.06.015

[9] Rosenberg AEBJ Fletcher CDMBJ Hogendoorn PCW Epithelioid haemangioma. WHO classification of tumours of soft tissue and bone IARC Press, 4th ed. Lyon, France: 2013.

[10] Nagai T Imai H Honda S Antiangiogenic effects of bisphosphonates on laser-induced choroidal neovascularization in mice. Invest Ophthalmol Vis Sci 2007;48:5716–21. 18055824 10.1167/iovs.07-1023

[11] Wood J Bonjean K Ruetz S Novel antiangiogenic effects of the bisphosphonate compound zoledronic acid. J Pharmacol Exp Therap 2002;302:1055–61. 12183663 10.1124/jpet.102.035295

[12] Marraoui W Michel JL Kemeny JL Imaging features of systemic cystic angiomatosis. Diagn Interv Imaging 2015;96:1211–3. 26141486 10.1016/j.diii.2014.08.009

[13] Marcucci G Masi L Carossino AM Cystic bone angiomatosis: a case report treated with aminobisphosphonates and review of the literature. Calcif Tissue Int 2013;93:462–71. 23836156 10.1007/s00223-013-9761-3

